# The Effects of Addition of Mononucleotides on Sma nuc Endonuclease Activity

**DOI:** 10.1100/2012/454176

**Published:** 2012-04-30

**Authors:** Julia Romanova, Maria Filimonova

**Affiliations:** Department of Microbiology, Kazan (Volga Region) Federal University, Kremliovskaya Street 18, Kazan 420008, Tatarstan, Russia

## Abstract

Examination of the effects of mononucleotides on Sma nuc endonuclease originated from Gram negative bacterium *Serratia marcescens* displayed that any mononucleotide produced by Sma nuc during hydrolysis of DNA or RNA may regulate the enzyme activity affecting the RNase activity without pronounced influence on the activity towards DNA. The type of carbohydrate residue in mononucleotides does not affect the regulation. In contrast, the effects depend on the type of bases in nucleotides. AMP or dAMP was classified as a competitive inhibitor of partial type. GMP, UMP, and CMP were found to be uncompetitive inhibitors that suggest a specific site(s) for the nucleotide(s) binding in Sma nuc endonuclease.

## 1. Introduction

Sma nuc endonuclease originated from Gram negative bacterium *Serratia marcescens* heads a broad range of homological nonspecific nucleases which widely spread in the world. Among them there is an apoptotic mitochondrial endonuclease Endo G.

Sma nuc is one of the most studied bacterial nucleases. Its structure, mechanism, physical, chemical, and biochemical properties are well known [1–11]. Controversially the mechanisms of Sma nuc regulation are insufficiently studied although it demonstrates a very potent digestive activity towards DNA and RNA resulting in mononucleotides production together with other nucleotides [12–14]. In particular, the published data on mononucleotides action are poor and incompatible [8, 12, 15] as well as mainly attributed to AMP, ATP, and DNA substrate although mononucleotides action on Sma nuc can perform a key mechanism of the enzyme regulation, by products. In accordance with the written above, the purpose of undertaken study was to examine in detail the effects of addition of mononucleotides on Sma nuc activity.

## 2. Materials and Methods

We used preparations of yeast DNA (“Sigma”, USA) and RNA (“US Biochemical Corporation”, USA). Sma nuc endonuclease (isoform Sm2) was isolated and characterized as previously shown [3, 16]. 

To study a direct influence of mononucleotides on DNase or RNase activity, aqueous solution of 5′AMP, 5′CMP, 5′GMP, 5′UMP (“Sigma”, USA), 5′dAMP, 5′dTMP, 5′dGMP, or 5′dCMP (“ICN”, USA) was added to the assay mixture at equimolar amount to the substrate concentration before addition of the enzyme. The activity was assayed by the described method [12, 13]. After addition of 13.1 nM Sm2 (0.35 *μ*g/mL) to 9-fold volume of assay mixture containing 50 mM Tris-HCl buffer, pH 8.5, 0.3 mM DNA or RNA, and 6 mM MgSO_4_, the incubation was performed at 37°C for 5–30 min so that about 15–50% of the substrate was converted to acid-soluble products. The hydrolysis was stopped with addition of chilled 4% perchloric acid. The precipitate was removed by centrifugation. The absorption of supernatant was monitored at 260 nm. Each experiment was repeated not less than 6 times. 

To carry out the inhibitory analysis, the nuclease activity was determined by the hyperchromic effect of hydrolysis of RNA using a *λ*-35 Perkin Elmer spectrophotometer. Apparent rates of the reaction were recorded until the progress curves became nonlinear. Rates were calculated from the linear part of the reaction progress curves (initial velocities) using the applied rate analysis software package. Experiments were carried out in 2 mm cuvettes at 37°C. The assay mixture contained 0.03 to 0.18 mM RNA, 50 mM Tris-HCl buffer, pH 8.5, from 1.2 to 7.2 mM MgSO_4_ and nucleotides when needed at concentration shown in the figure captions. After addition of 1.79 *μ*M isoform Sm2 (47.83 *μ*g/mL) to 100-fold volume (500 *μ*L) of prewarmed (3–5 min) assay mixture, change in absorbance at 260 nm was recorded immediately. The reaction velocities were expressed in KU. Concentration of the isoform Sm2 was calculated based on the molecular mass and molar extinction coefficient of 47.292 M^−1^·cm^−1^ [10]. Concentration of RNA in nucleotide equivalents was calculated using *ε*_260_ of 6500 M^−1^·cm^−1^ [17]. Inhibitor constants for CMP, GMP, and UMP were determined from plots of *S*/*V* against [*I*] as a function of the substrate concentration by the method of Cornish-Bowden [18], for AMP and dAMP by building a double reciprocal plots of dependency of the length of segments obtained in Lineweaver-Burk plots on the inhibitor concentrations [19]. 

## 3. Results and Discussion

In accordance with previously formed principles, the examination was carried out at the enzyme/substrate ratio of 1.3–3.2 pmol/mg [20] and Mg^2+^ per phosphate in substrate of 20–40 : 1 [6]. To maintain the constant ratio of Mg^2+^ per substrate phosphate Mg^2+^ was added to the solution at equimolar to mononucleotide concentration as deviation from the optimal ratio of Sma nuc/substrate/magnesium cations [6, 20] affects the enzyme activity. 

**Figure 1:**
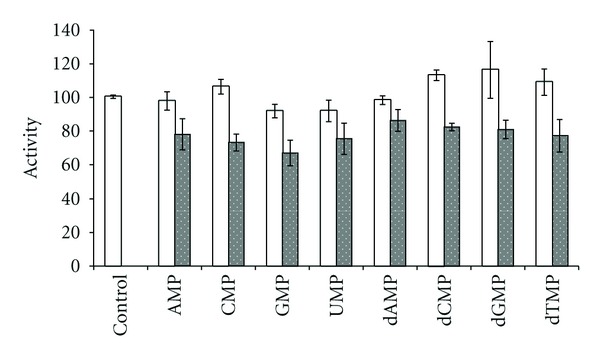
The activity (%) toward DNA (empty area) or RNA (doted area) substrate in the presence of the 0.3 mM nucleotide. Here and later a control is the activity in the absence of nucleotides (taken as 100%).

**Figure 2:**
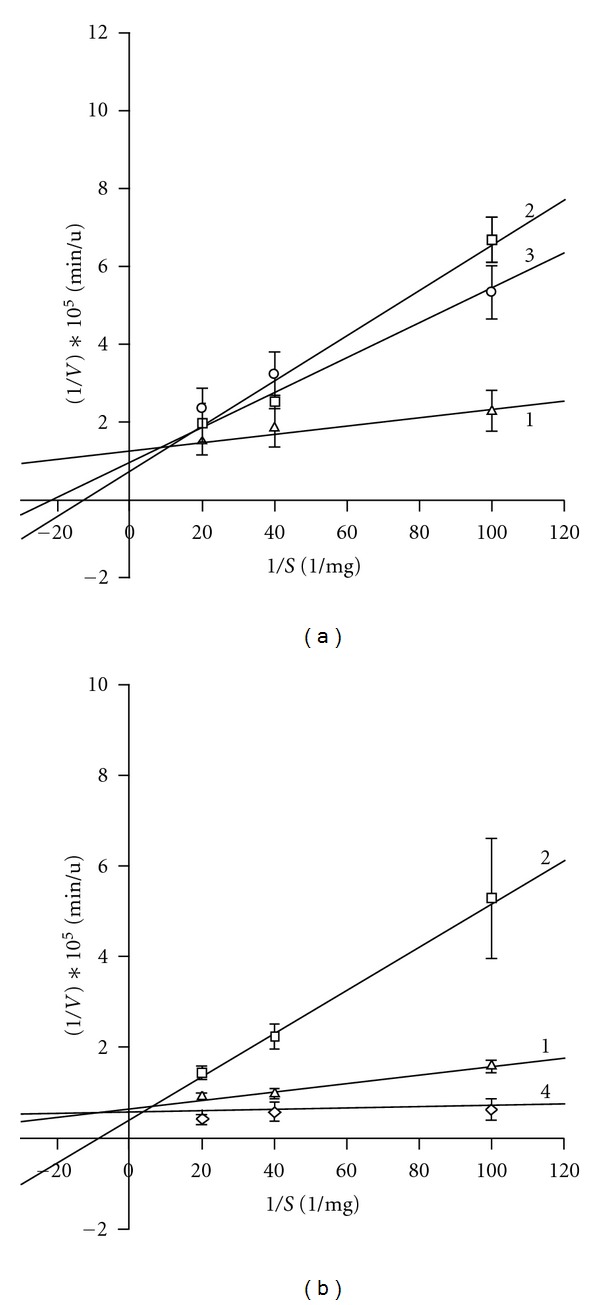
A dependence of the initial reaction rate on the substrate concentration in the presence (lines 2–4) and in the absence (line 1) of AMP (a) or dAMP (b). 1: 0 mM (control), 2: 0.29-, 3: 0.58-, 4: 1.2 mM mononucleotide.

**Figure 3:**
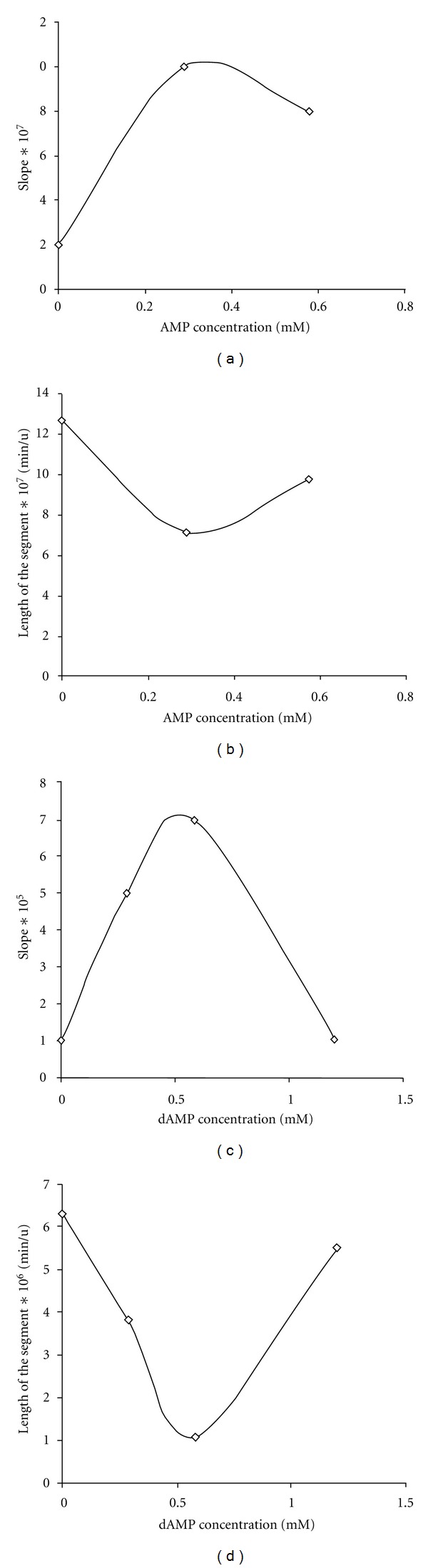
A dependence of the slopes (a, c) and the segments cut off at the ordinate axis (b, d) in reciprocal plots from Figure 2 on AMP (a, b) or dAMP (c, d) concentrations.

**Figure 4:**
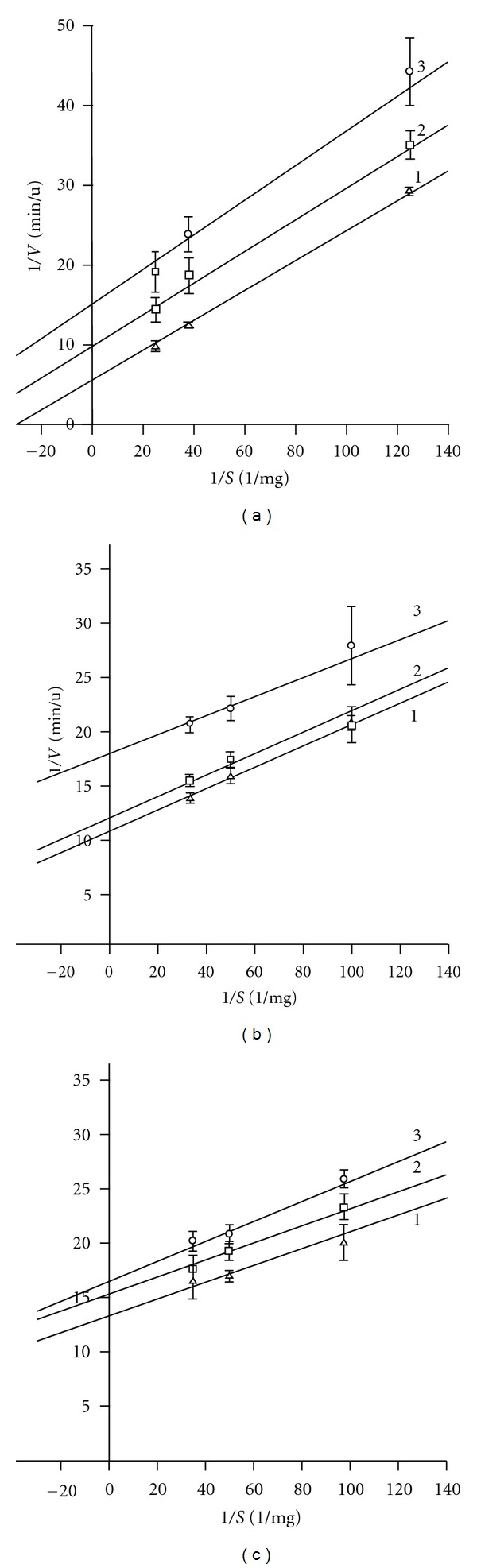
A dependence of the initial reaction rate on the substrate concentration in the presence (lines 2-3) and in the absence (line 1) of CMP (a), GMP (b), and UMP (c). 1: 0 mM (control), 2: 0.29-, 3: 0.58 mM mononucleotide.

The results presented in Figures 1–4 and Table 1 demonstrate a reality of regulation of Sma nuc activity by products. In particular the data show 1.2-1.3 fold decrease of the activity towards RNA (Figure 1) that is observed upon addition of mononucleotides independently on the type of either carbohydrate residues or the bases. The data also suggest a lack of the influence on the DNase activity as the nucleotides action on Sma nuc activity towards DNA is not authentic.

Determining the type of inhibition shows a lack of pronounced difference between AMP and dAMP. Graphical representation of the double reciprocal plots (Figure 2) of the activity depending on RNA concentration at fixed concentrations of AMP and d AMP partly reminds the competitive type of inhibition and reveals a complicated mechanism of the regulation. In particular, as shown in Figure 2, the slope of the curves increases in the presence of AMP or dAMP, except for 1.2 mM dAMP, if to compare with curves in the absence of nucleotides, and controversially decreases with rising the concentration to 0.58 or 1.2 mM, if to compare with 0.29 mM AMP or dAMP.

Except for the curve obtained in the presence of 1.2 mM dAMP, other curves intersect near the ordinate axis at the points lying above the *x*-axis, in its positive values. The curve obtained in the presence of 1.2 mM dAMP looks almost parallel to the axis 1/*S*. The point of intersection of the curve with the line of control is located almost on the 1/*V* axis. Direct correlation between increase in the slope of the curves and the amount of nucleotides in the medium is not observed. The analysis of secondary curves (Figure 3) which are plots of the slopes from the reciprocal plots shown in Figure 2, and the segments cut off at the ordinate axis by these curves, reveals the next.

Independently on type of the added nucleotide, the shape of plots for the slopes reminds a convex parabola and for the segments a concave parabola that in respect with Cleland's classification corresponds to a hyperbolic activation or hyperbolic inhibition of the enzyme [21]. It suggests that upon addition of AMP or dAMP a partially competitive inhibition occurs. At this case AMP or dAMP forming a complex with Sma nuc does not completely prevent the RNA binding and reduces the enzyme affinity to RNA substrate in competition with RNA for the binding site. This results in producing the alternative ternary complex (EI + *S* = ESI) which at certain nucleotide concentrations dissociates faster than in regular way (EIS = EI + *P*) that is followed with increasing enzymatic rate.

Similar values of inhibitor constants for AMP and dAMP (Table 1) confirm our assumption on the lack of difference between AMP and dAMP in Sma nuc regulation, in particular a lack of influence of carbohydrate residues in nucleotides on the pattern of inhibition. In this connection, the further inhibitory analysis was carried out with ribonucleotides.

A comparative analysis of Sma nuc inhibition with GMP, UMP, and CMP has revealed their self-similarity and distinction from AMP and dAMP.

As shown in Figure 4, double reciprocal plots as a function of concentrations of CMP, GMP, and UMP resemble straight lines that are parallel to the line obtained in the absence of nucleotides. This kind of plots is indicative to the uncompetitive inhibition that is usually observed in single substrate reaction [22] when the inhibitor binds only to enzyme-substrate complex.

Determining the inhibitor constants (Table 1) confirmed a reminded above difference between AMP or d AMP and other inspected nucleotides, especially GMP or UMP. The value of CMP inhibitor constant was close to the constants of AMP or d AMP.

Thus, examination of the effects of mononucleotides on Sma nuc displayed that any mononucleotide produced by Sma nuc during hydrolysis of DNA or RNA may regulate the enzyme activity affecting the RNase activity without pronounced influence on activity towards DNA. The type of carbohydrate residues in mononucleotides does not affect the regulation. In contrast the effects depend on the type of bases in nucleotides. AMP or dAMP classified as a competitive inhibitor of partial type was found to bind the enzyme prior to the enzyme binding with the substrate and to shear the binding site with the substrate that can both inhibit and activate the enzyme. In contrast, GMP (dGMP), UMP (dUMP), and CMP (dCMP) classified as uncompetitive inhibitors are not able to cooperate with the enzyme prior to formation of the enzyme-substrate complex and bind to specific binding site(s) which becomes available only after formation of the enzyme-substrate complex.

## Figures and Tables

**Table 1:** The inhibitor constants.

Nucleotide	Ki, mM
AMP	0.0765
dAMP	0.0600
CMP	0.0862
GMP	0.229
UMP	0.3107
